# Competition between Visual Events Modulates the Influence of Salience during Free-Viewing of Naturalistic Videos

**DOI:** 10.3389/fnhum.2016.00320

**Published:** 2016-06-28

**Authors:** Davide Nardo, Paola Console, Carlo Reverberi, Emiliano Macaluso

**Affiliations:** ^1^Neuroimaging Laboratory, Santa Lucia FoundationRome, Italy; ^2^Institute of Cognitive Neuroscience, University College LondonLondon, UK; ^3^Department of Psychology, University of Milano-BicoccaMilan, Italy; ^4^NeuroMi—Milan Center for Neuroscience, University of Milano-BicoccaMilan, Italy; ^5^Impact Team, Lyon Neuroscience Research CenterLyon, France

**Keywords:** spatial attention, eye movements, real world, salience, competition, fMRI, MVPA

## Abstract

In daily life the brain is exposed to a large amount of external signals that compete for processing resources. The attentional system can select relevant information based on many possible combinations of goal-directed and stimulus-driven control signals. Here, we investigate the behavioral and physiological effects of competition between distinctive visual events during free-viewing of naturalistic videos. Nineteen healthy subjects underwent functional magnetic resonance imaging (fMRI) while viewing short video-clips of everyday life situations, without any explicit goal-directed task. Each video contained either a single semantically-relevant event on the left or right side (Lat-trials), or multiple distinctive events in both hemifields (Multi-trials). For each video, we computed a salience index to quantify the lateralization bias due to stimulus-driven signals, and a gaze index (based on eye-tracking data) to quantify the efficacy of the stimuli in capturing attention to either side. Behaviorally, our results showed that stimulus-driven salience influenced spatial orienting only in presence of multiple competing events (Multi-trials). fMRI results showed that the processing of competing events engaged the ventral attention network, including the right temporoparietal junction (R TPJ) and the right inferior frontal cortex. Salience was found to modulate activity in the visual cortex, but only in the presence of competing events; while the orienting efficacy of Multi-trials affected activity in both the visual cortex and posterior parietal cortex (PPC). We conclude that in presence of multiple competing events, the ventral attention system detects semantically-relevant events, while regions of the dorsal system make use of saliency signals to select relevant locations and guide spatial orienting.

## Introduction

In any everyday life situation the sensory system receives more information from the outside world than the brain can fully process, with many objects and events that compete for the limited processing resources. Spatial attention control allows focussing on signals located in one region of space, selecting relevant information for in-depth processing (Desimone and Duncan, 1995). Both sensory characteristics of the external stimuli, and internal signals related to current goals, intentions, and expectations contribute to spatial orienting control (Posner, 1980; Yantis, 2000). Here, using short video-clips of real-world scenes we investigated how the competition between visual events affects overt spatial orienting in the absence of any explicit goal-directed task, and how such competition modulates the impact of stimulus salience on orienting behavior and brain activity.

Behaviorally, the issue of competition in spatial attention has been often addressed using visual search tasks. Subjects are required to search for a given target (e.g., a letter) within a visual array containing many distractors that compete for processing (i.e., non-target letters). When the target is characterized by a very distinctive feature (e.g., a red target-letter among green distractor-letters), the target seems to “pop-out” of the visual array, the detection is fast and the search times are largely independent of the number of distractors (efficient search). In this situation, the sensory characteristics of the visual input guide the selection process and attention is attracted automatically towards the salient pop-out target (stimulus-driven control, Theeuwes, 1992; Yantis, 1993). By contrast, when a target is defined by a combination of features, some of which are shared with distractors, the search is slow and dependent on the number of distractors (inefficient search). In this case, to solve the competition between the target and the distractors, spatial attention is shifted sequentially between the display items until the target is found, or all the items have been classified as distractors (Treisman and Gelade, 1980; Treisman, 1986; but for criticisms, see Wolfe et al., 1989; Cave and Wolfe, 1990). The selection process is governed primarily by internal knowledge about the target-defining features and attention is controlled in a voluntary manner (goal-directed control, Bacon and Egeth, 1997; see also Duncan and Humphreys, 1989, 1992).

Non-invasive imaging studies provide us with extensive evidence about the neurophysiological bases of visuospatial control in humans (for reviews, see Corbetta and Shulman, 2002; Vossel et al., 2014). For example, Kastner et al. (1999) investigated how voluntary control of spatial attention influences the processing of competing visual stimuli. Using functional magnetic resonance imaging (fMRI), the authors showed that the simultaneous presentation of several irrelevant stimuli in one visual quadrant lead to the suppression of the corresponding functional responses in the visual cortex, compared to the sequential presentation of the same stimuli (“competitive interactions”). However, when the participants directed voluntary attention to the stimulated quadrant such interactions were reduced (cf. “Biased Competition Theory” of selective attention, Desimone and Duncan, 1995). Besides these effects of competition in the visual cortex, the same study also highlighted the involvement of the dorsal frontoparietal cortex that is assumed to control goal-directed, voluntary attention (Kastner and Pinsk, 2004; Pinsk et al., 2004; Foley et al., 2012). On the other hand, regions in the right inferior frontoparietal cortex (i.e., the temporoparietal junction, R TPJ; and the inferior frontal gyrus, R IFG) have been associated with stimulus-driven control. These regions activate when participants are presented with isolated targets at an unattended location (e.g., invalid trials in spatial cueing tasks; Arrington et al., 2000; Corbetta et al., 2000). Related to the issue of attentional selection between competing stimuli, Serences et al. (2005) showed activation of the R TPJ in response to peripheral distractors while participants performed a central detection task, but only when the distractors contained some goal-related, task-relevant target-defining feature (i.e., distractors of the same color as the target).

These and many other imaging studies provide us with important insights about brain regions involved in voluntary and stimulus-driven attention, but the vast majority of these studies involved simple stimuli and very specific, goal-directed tasks. Typically, participants are instructed to detect or discriminate a given target item (e.g., a letter or some other simple shape) and—for hundreds of trials—they will process the incoming visual input with the sole scope of detecting/discriminating that specific stimulus. This approach allows controlling for many experimental factors, such as the number, size and position of the stimuli. However, it should be considered that attention control in real-life has to face a rather different set of constraints (e.g., see Wolfe et al., 2011; Peelen and Kastner, 2014). In the real world the attentional system has to deal with highly complex, variable and dynamic sensory input. Further, while both stimulus-driven and goal-related signals contribute to attention control (see below), we seldom explore the environment with the sole scope of detecting one specific object or event: that is, following some specific task-instructions. Instead, we have a vast amount of prior internal knowledge about the spatial layout of objects in the real world, which provides us with additional information for the allocation of spatial attention (Castelhano and Heaven, 2011; Spotorno et al., 2014; Wu et al., 2014).

One approach to investigate attention control in more realistic, life-like conditions is to utilize pictures or videos of real-world scenes (e.g., Carmi and Itti, 2006; Summerfield et al., 2006; Einhäuser et al., 2007; Nardo et al., 2011, 2014; Malcolm and Shomstein, 2015; Ogawa and Macaluso, 2015; for a review, see also Peelen and Kastner, 2014). Many studies have now demonstrated that both the sensory characteristics of the visual input, as well as internal information (such as task-related goals, prior knowledge and expectations) contribute to the processing of such complex stimulus material. Stimulus-driven signaling can be investigated using computational models that seek to extract salient locations in the image (e.g., “saliency maps” proposed by Itti et al., 1998; Itti and Koch, 2001). Saliency maps characterize the stimulus-driven contribution to spatial orienting and have been shown to successfully predict patterns of eye movements during free-viewing of complex scenes (Carmi and Itti, 2006; Elazary and Itti, 2008) and to modulate brain activity associated with spatial attention (e.g., Bogler et al., 2011; Nardo et al., 2011; Santangelo and Macaluso, 2013). At the same time, higher-level factors strongly influence spatial orienting in natural scenes. When participants are asked to search for a target-object defined by specific task-instructions, prior knowledge about where that object can be usually found makes the search more efficient (Evans et al., 2011; Greene and Wolfe, 2011; Wolfe et al., 2011), and the discrimination between targets and distractors is largely dependent on semantic knowledge (Peelen et al., 2009; Draschkow et al., 2014; Seidl-Rathkopf et al., 2015). These internal factors can influence scanpaths (Humphrey and Underwood, 2009; Malcolm and Henderson, 2010) and reduce the impact of stimulus-driven signals during overt exploration of real-world scenes (Einhäuser et al., 2008; Stoll et al., 2015). Nonetheless, it should be noted that most of the previous studies that investigated spatial attention with naturalistic stimuli made use of explicit goal-directed tasks (e.g., search for a specific target-object within real scenes, Neider and Zelinsky, 2006; Castelhano and Heaven, 2010; Malcolm and Henderson, 2010; see also Santangelo et al., 2015), which is likely to influence the neural systems involved in control operations (see section above about regions associated with goal-directed attention).

Here we investigated the role of stimulus-driven signals for spatial orienting in natural scenes, specifically in the absence of any explicit task. In a previous fMRI study that involved free-viewing of a virtual visual environment (i.e., without any goal-directed task), we reported activation of the dorsal frontoparietal network when stimulus-driven signals successfully attracted subjects’ gaze/attention (Nardo et al., 2011; “efficacy” of salience for spatial orienting). The same study also showed that semantically-relevant, gaze/attention-grabbing events (moving avatars, in that study) were processed in the ventral attention system, indicating a possible dissociation between the dorsal and the ventral frontoparietal systems when orienting attention within complex visual environments, in the absence of any explicit goal-directed task. In the current study, we extend this line of investigation by specifically enquiring how competing visual events are processed in the attention control networks, and how such competition interacts with stimulus-driven (salience) signaling.

**Figure 1:**
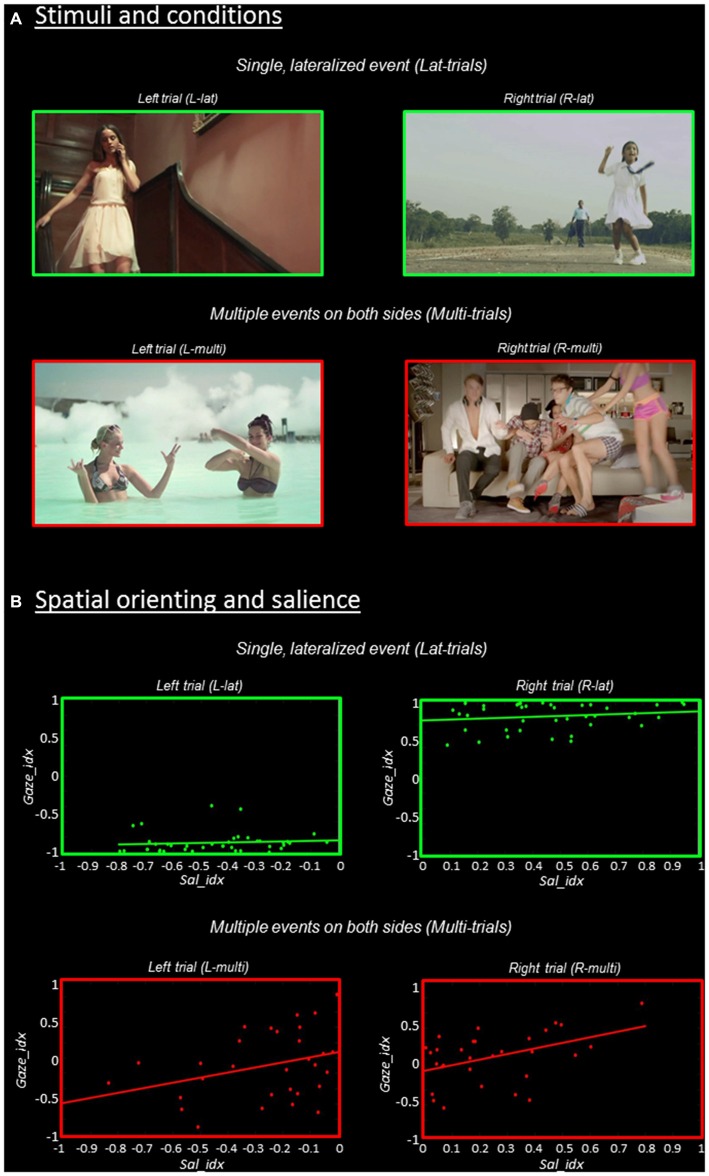
**Stimuli and behavioral results. (A)** Stimuli and experimental conditions. Single frames extracted from the videos that contained a single, lateralized visual event are shown in the upper panel (“Lat-trials”, green borders); while frames extracted from the videos including multiple events on both sides are shown in the lower panel (“Multi-trials”, red borders). The further categorization in “Left” vs. “Right” trials was done based on saliency maps (cf. “Materials and Methods” Section). **(B)** Relationship between spatial orienting (Gaze_idx) and stimulus salience (Sal_idx), separately for the four experimental conditions. The Gaze_idx correlated with Sal_idx only in trials containing multiple competing events (“Multi-trials”, plots in red), while in Lat-trials the side of the main visual event largely determined spatial orienting (cf. plots in green). For both Gaze_idx and Sal_idx positive values indicate a rightward bias, while negative values indicate a leftward bias.

In the current fMRI study, participants were presented with short (1.5 s) video-clips that included either multiple semantically-relevant events on both sides of space (Multi-trials: strong competition), or a single main event lateralized on the left or right hemifield (Lat-trials: weak competition; for a few examples, see Figure 1A). Participants were asked to simply watch the videos and were allowed to move their eyes, that is, a naturalistic free-viewing situation without any explicit goal-directed task. For each stimulus, we quantified the strength of the stimulus-driven signals using saliency maps (Itti et al., 1998). We created a salience lateralization-index (Sal_idx) as the difference between the salience in the two hemifields. In order to index to what extent each stimulus was able to drive spatial attention to either side of space we used eye-movements recorded during the viewing of the videos. We computed a gaze lateralization index (Gaze_idx) as the difference in time the participants spent looking towards the two hemifields.

Behaviorally, we assessed the contribution of stimulus-driven signals to spatial orienting testing for the relationship between the salience index and the gaze index, separately for videos with multiple vs. single-lateralized events. We predict that in Lat-trials participants would systematically orient towards the side of the single semantically-relevant event (cf. Einhäuser et al., 2008; Nuthmann and Henderson, 2010; Stoll et al., 2015). If so, the “video-specific” level of lateralization of salience will have little effect on the time spent looking towards the relevant hemifield, without any correlation between the saliency and gaze indexes for these trials. By contrast, we expect that stimulus-driven salience would contribute to spatial orienting in Multi-trials, when the competition between co-occurring visual events will reduce any spatial orienting bias associated with the distinctive events. Now, videos with high levels of salience lateralization should lead to greater gaze-orienting towards the most salient hemifield, compared with Multi-trials entailing low levels of salience lateralization. The latter would result in a significant correlation between the saliency and the gaze indexes in Multi-trials.

For the analyses of imaging data, we first tested for the overall effect of competition between distinctive events by directly comparing Multi-trials vs. Lat-trials. We used both standard univariate methods and multivariate pattern analysis (MVPA). The latter has several advantages over univariate methods (Norman et al., 2006) and is gaining increasing interest in cognitive neuroscience (for reviews, see Mahmoudi et al., 2012; Serences and Saproo, 2012; Haynes, 2015). Recently, we have shown that MVPA can unveil the role of specific nodes of the attentional system (i.e., processing of invalid trials in the left TPJ), which would be missed using traditional subtraction methods (Silvetti et al., 2015; for recent applications of MVPA in studies with naturalistic stimuli, see also Bogler et al., 2011; Epstein and Morgan, 2012; Preston et al., 2013; Johnson and Johnson, 2014; Linsley and MacEvoy, 2014; Watson et al., 2014).

We formulated two main hypotheses. On the one hand, if the high levels of competition in Multi-trials is handled via sequential orienting towards the multiple visual events, we would anticipate the engagement of the ventral attention system, which we previously associated with the detection of distinctive events in free-viewing conditions (Nardo et al., 2011). Further, if stimulus-driven signaling takes effect to solve competition in Multi-trials (cf. also section of behavioral predictions, above), we may expect the engagement of the dorsal attention system that have been found activated when visual salience is used to guide spatial orienting and attentional selection (Bogler et al., 2011; Nardo et al., 2011; see also Santangelo and Macaluso, 2013). The further characterization of the response of the frontoparietal attention systems in these naturalistic conditions will contribute to bridge the gap between traditional experiments in the laboratory and brain functioning in the real world (Hasson et al., 2004; Peelen and Kastner, 2014).

## Materials and Methods

### Subjects

This study is part of a larger research project about spatial attention control in people who suffered from stroke to the right hemisphere (neglect patients). In this framework, we recruited a cohort of healthy controls who will be later compared with neurological patients. Here, we report the data of 19 healthy aged volunteers (i.e., all the healthy subjects who participated in the stroke project; age range: 44–81 years, mean age: 62 ± 12.1; 10 males and 9 females). Their inclusion criteria were: right-handedness, no history of psychiatric or neurological disease or drug abuse, and normal or corrected-to-normal (contact lenses) visual acuity. After having received instructions, all participants gave their written informed consent. The study was approved by the independent Ethics Committee of the Santa Lucia Foundation (Scientific Institute for Research, Hospitalization and Health Care).

### Stimuli and Salience Index

The set of stimuli consisted of 140 videos showing everyday life situations. We obtained a collection of non-Italian TV commercial clips, partly purchased from an Advertising Archive^1^ and partly downloaded from YouTube (see Figure 1A for a few screen shots). Using a video editing software (Final Cut Pro, Apple Inc., Cupertino, CA, USA), we selected 1.5 s video-segments that included a single continuous scene with either one lateralized distinctive event (Lat-trials) or multiple events in both hemifields (Multi-trials). Most of the distinctive, semantically-relevant events consisted in one or more persons in the foreground, who either performed an action (walking, dancing, manipulating objects, etc.) or changed posture. In approximately 10% of videos, the event consisted in a moving vehicle (plane, car, motorbike, etc.; equally distributed across conditions). We discarded any segments that included writings, which allowed us to left-right flip the videos as an additional experimental control (cf. the experimental factor of “left/right”, below). Stimuli were presented in a randomized order, balancing the video flipping across subjects. The inter-trial intervals ranged from 3 to 7.4 s (mean 5.2). During the inter-trial interval a fixation point was presented in the center of the screen.

For each stimulus, we indexed the strength of stimulus-driven signals using saliency maps (Itti et al., 1998; Itti and Koch, 2001). For each frame, we obtained a saliency map based on multi-scale contrasts of intensity, color, orientation, motion and flicker, as computed using the MT_TOOLS toolbox.^2^ Next, for each video we generated a single value (Sal_idx) that represents the level of lateralization of the saliency signals. The Sal_idx was computed by averaging the saliency values separately in the left and the right side of each map (excluding a central region of 2°, cf. also “gaze index” below) and then averaging these values over the entire stimulus duration. Finally, the level of salience lateralization was computed as a single normalized coefficient Sal_idx = (L − R)/(L + R), with negative/positive values indicating greater salience on the left/right side of the video (see also Nardo et al., 2014).

The Sal_idx was used for both behavioral analyses—that is, to evaluate the contribution of stimulus-driven signaling for gaze-orienting (cf. Gaze_idx, below)—and for correlational analyses using fMRI BOLD time-series. In addition, the Sal_idx allowed us to categorize each stimulus as either “Left” or “Right”, which was included as a factor in the modeling of the fMRI data (L- vs. R-trials), together with the main factor concerning the level of competition (Multi- vs. Lat-trials). The full set of videos included 80 lateralized trials and 60 multiple trials, equally split into left- and right-trials.

In addition, we examined the saliency maps to gain some further insight about the spatial organization of the scenes in the two main conditions (Multi- and Lat-videos). Despite saliency maps are based on local discontinuities in low-level features (e.g., color, orientation, motion, etc.), previous work showed that such discontinuities tend to correspond to “interesting objects” in the scene (Elazary and Itti, 2008). Therefore, here we predicted that the Multi-trials should include more saliency clusters as compared with Lat-trials. For each video, we computed the number of saliency clusters by adding the saliency maps associated with each video-frame and counting the number of saliency cluster per video (i.e., continuous patches of pixels with saliency values larger than zeros). We compared the number of saliency clusters in Multi- vs. Lat-trials using a two-sample *t*-test. With these 2D maps we also further quantified the overt orienting behavior of the participants, by asking the additional question of whether the likelihood to fixate a salient location/cluster changed as a function of Multi- vs. Lat-conditions. For each video, we computed the percentage of fixations falling inside the saliency clusters and compared these values between Multi- vs. Lat-trials using a two-sample *t*-test.

### Eye-Movements and Gaze Index

Subjects viewed the 140 videos twice, first in a behavioral session outside the MR scanner and then 1 week later during the fMRI experiment. Behavioral data were collected as part of the research project that included comparisons with brain-damaged patients (cf. also “Subjects” section, above). The behavioral session was carried out in a quiet, dimly lit room. Subjects seated in front of a laptop equipped with a portable eye-tracking system operating at 120 Hz (RED-m Eye Tracking System 3.2; SensoMotoric Instruments^3^), at a distance of approximately 60 cm from the screen. Subjects were simply asked to freely-view the stimuli. During the fMRI sessions, gaze-position was recorded with a long-range MR-compatible eye-tracking system operating at 60 Hz (Applied Science Laboratories, Bedford, MA, USA; Model 504). The videos were back-projected onto a screen at the back of the MR bore, visible to the subjects via an MR-compatible mirror. The horizontal visual angle was approximately 24° pre-scanning, and *ca.* 19° in-scanner. The eye-tracking data were processed with custom scripts using MT_TOOLS^4^. Fixations were defined as gaze-position remaining within an area of 1.5° × 1.5° for a minimum duration of 100 ms.

In order to obtain the most accurate indexes of orienting efficacy, we imposed several constraints to select the eye-tracking data that were included for the computation of the video-specific Gaze indexes. For each subject and each trial/video, we considered only eye-traces where the pre-stimulus gaze-position was within ±2° of the center of the screen and at least 50% of data-points during the presentation of video could be categorized as fixations (i.e., excluding trials including many blinks and/or other artifacts). We counted how many trials satisfied these criteria and selected participants who had at least six trials for each of the four experimental conditions. The number of participants who fitted these criteria was 11 for data acquired during scanning vs. 16 in the pre-scanning session. Therefore, for the computation of the Gaze_idx we used the eye-tracking data of 16 participants in the pre-scanning session (cf. also Nardo et al., 2011; where we validated the use of eye-movement data collected outside the scanner to compute indexes of spatial orienting). Note that the pre- vs. during-scanning Gaze_idx were highly correlated (*r* = 0.92; *p* < 0.001).

The Gaze_idx represents the efficacy of each video to generate a systematic shift of overt attention towards either side. For each video, the index was computed as the proportion of time that participants spent looking towards the two hemifields, that is, (Ltime − Rtime)/(Ltime + Rtime) (see Nardo et al., 2014). Since any small deviation of horizontal gaze-position around the center of the screen (even below the spatial precision of gaze measurement) would strongly affect this index, the gaze data-points falling within ±2° of the center of the screen were not considered for the computation of the L/R-time values.

In addition, for each video we computed the number of saccades that the 16 participants made during free-viewing of the videos in the pre-scanning session. For each video, the number of saccades was defined as the number of fixations (see above) minus one. Under the hypothesis that the presence of multiple distinctive events would lead to sequential exploration of the different events, we compared Multi- vs. Lat-trials expecting a larger number of saccades in the former.

### fMRI Data Acquisition and Preprocessing

A Siemens Allegra (Siemens Medical Systems, Erlangen, Germany) 3 T scanner equipped for echo-planar imaging (EPI) was used to acquire fMRI. A quadrature volume head coil was used for radio frequency transmission and reception. Head movement was minimized by mild restraint and cushioning. Thirty-two slices of fMRI were acquired using blood-oxygen-level dependent (BOLD) imaging (3 × 3 mm, 2.5 mm thick, 50% distance factor, repetition time (TR) = 2.08 s, echo time (TE) = 30 ms), covering the entirety of the cortex. We also acquired a Magnetization-Prepared Rapid Gradient-Echo (MPRAGE) sequence as an anatomical reference (TR = 2.5 s, TE = 2.74 ms, voxel size 1 × 1 × 1 mm, matrix resolution 256 × 256 × 176, axial acquisition). Each subject underwent two fMRI runs, each including 230 volumes. Data preprocessing was performed with SPM12b (Wellcome Department of Cognitive Neurology). The first four volumes of each run were discarded to allow T1 saturation effects. Images were first manually realigned along the anterior commissure-posterior commissure (AC-PC) axis, then realigned, unwarped, and slice-timed with the middle slice as a reference.

### Standard Univariate Analysis

Standard univariate fMRI analysis was performed with SPM12b, and included first- and second-level analyses. Stimuli were modeled as delta functions (duration = 1.5 s, corresponding to the duration of the videos), convolved with the standard hemodynamic response function (HRF). The first-level model included four conditions, obtained by combining the factors “competition” (Multi- vs. Lat-trials) and “side” (L- vs. R-trials). All models included the head-motion realignment parameters as additional covariates of no interest. The time series were high-pass filtered at 128 s and pre-whitened by means of autoregressive model AR(1). Linear contrasts were used to average the parameter estimates across the two fMRI-runs, separately for the four conditions of interest (Lmulti, Rmulti, Llat, Rlat). To allow for group-level analyses, the resulting four contrast images per subject were normalized to the Montreal Neurological Institute (MNI) space. The normalization parameters were estimated from the (co-registered and segmented) individual T1 volume, re-sampled (3 × 3 × 3 mm) and smoothed with a Gaussian kernel (Full width at half maximum, FWHM = 8 × 8 × 8 mm), including the SPM12b brain-mask as an explicit mask.

For second-level group analysis, a flexible factorial design modeled the four conditions of interest (Lmulti, Rmulti, Llat, Rlat), plus the main effect of subjects. Sphericity correction was applied to account for any non-independent error term for repeated measures and any difference in error variance across conditions (Friston et al., 2002).

We report the main contrast of interest that directly compared the conditions with multiple events (“Multi”, high competition) vs. conditions with a single distinctive event (“Lat”, low competition), averaging across the factor of side. The level of significance was set to *p*-Family-wise error (FWE)-corr. = 0.05, cluster-level corrected for multiple comparison (cluster size estimated at *p*-unc. = 0.001). We also report some effects at a lower *p*-unc. < 0.001, with the aim of evaluating the results of the univariate analysis against the results of the multivariate MVPA approach (see below, and Table 1).

**Table 1:** **Results of the univariate and multivariate analyses (MVPA; cross-validation)**.

	Univariate analysis (Multi > Lat)				Multivariate analysis (Multi vs. Lat)				
	Cluster		Peak		Cluster		Peak		
Region	*p*	*k*	*t*	*x, y, z*	*p*	*k*	*t*	*x, y, z*	% acc
R calcarine	<0.001	1287	6.85	15 −88 5	<0.001	3637	7.01	12 −79 11	65.4
R lingual			7.46	18 −70 −7			5.99	27 −67 −4	58.1
L calcarine			7.14	−9 −88 2			5.96	−9 −85 −4	60.7
L lingual			7.54	−9 −79 −10			6.19	−12 −79 −1	62.0
L/R PCN	0.008	114	5.12	3 −55 50			6.96	6 −61 56	61.3
R LOC	*0.408*	*30*	*4.40*	*45* −64 17			4.61	48 −70 2	62.1
L LOC	*0.731*	*17*	*4.24*	*−51 −76 5*			7.09	−48 −73 2	59.9
R SOC	–	–	–	–			5.85	30 −67 29	62.7
L SOC	–	–	–	–			5.62	−21 −73 29	60.1
R TPJ	*0.621*	*21*	*4.11*	*54 −52 23*			4.14	48 −58 20	61.9
R STS	–	–	–	–	0.001	97	5.65	48 −28 −13	51.4
R IFG/MFG	–	–	–	–	<0.001	194	5.19	36 5 26	54.0

*Multi: videos with multiple events; Lat: videos with a single lateralized event. Cluster: FWE-corrected p-value and cluster size (k = number of voxels). Peak: t-statistics and x, y, z-coordinates in MNI space. % acc: average accuracy of the MVPA. Regions: R/L, right/left hemisphere; PCN, precuneus; LOC, lateral occipital cortex; SOC, superior occipital cortex; TPJ, temporoparietal junction; STS, superior temporal sulcus; IFG/MFG, inferior/middle frontal gyri. In italics: areas fully significant in MVPA, but activated only at uncorrected p-values (p < 0.001) in the univarate analysis*.

### Multi-Voxel Pattern Analysis

Multivariate analyses were performed with the Decoding Toolbox^5^. At the subject-level, with unnormalized and unsmoothed data, we performed MVPA, using a Linear Support Vector Machine (LSVM) classifier with a 6-fold cross-validation procedure. We used the stimulus/video conditions as classes and the parameter estimates (beta-images) derived from individual fMRI first-level analyses as features. For the MVPA analysis the first-level models were re-constructed now including three separate regressors for each of the four condition (Lmulti, Rmulti, Llat, Rlat). Specifically, for each fMRI-run the trials belonging to each condition were pseudo-randomly split into three sub-groups. The pseudo-randomization took into account the possible influence of the previous stimulus by ensuring, for each trial, that the previous trial belonged to one of the four conditions with equal probability. Moreover, we balanced the overall temporal distribution of the trials in the three sub-groups by balancing the number of trial-onsets from the first and the second half of the run. With this, for each fMRI-run, we obtained three regressors modeling the two Lat-conditions (including 6 or 7 trials) and three regressors modeling the Multi-conditions (5 trials per regressor). Accordingly, the new first-level models included 12 regressors of interest per run, which—over the two runs—enabled us to obtain six parameter estimates (beta-images) for each of the four conditions of interest.

These 24 beta-images were divided into six image-sets, with each set containing one beta-image per condition (i.e., Llat, Rlat, Lmulti, Rmulti). To avoid over-fitting and biased accuracy estimation, we applied a leave-one-out cross-validation procedure. A classifier was trained to discriminate between Multi (Lmulti, Rmulti) and Lat (Llat, Rlat) conditions using five of the six image-sets available. The classifier was then tested on the last remaining set. Thus, for each run of cross-validation, the classifier was tested over four beta-images, one for each of the four experimental conditions (Llat, Rlat, Lmulti and Rmulti).

The MVPA was performed with a searchlight approach (sphere with a radius of four voxels). The searchlight considered only gray-matter voxels. The latter were identified using the individual T1 volume that was segmented, smoothed (FWHM = 4 × 4 × 4 mm), re-sampled to the EPI resolution (3 × 3 × 3.75 mm) and thresholded to identify the voxels belonging to the gray matter (probability threshold = 10%). For each subject, the MVPA produced an accuracy map including the decoding accuracy above chance (i.e., accuracy minus 50%).

For statistical inference at the group-level, these accuracy maps were normalized to the MNI space. The normalization parameters were estimated from the (co-registered and segmented) individual T1 volume, re-sampled (3 × 3 × 3 mm) and smoothed with a Gaussian kernel (FWHM = 4 × 4 × 4 mm), including the SPM12b brain-mask as an explicit mask. Statistical inference was obtained with a one-sample *t*-test that assessed where the classification accuracy was larger than chance using between-subjects variance. The level of significance was set to *p*-FWE-corr. = 0.05, cluster-level corrected for multiple comparison (cluster size estimated at *p*-unc. = 0.001).

### Parametric Modulations Analyses

We used the Sal_idx and the Gaze_idx to identify any brain region where activity was modulated according to stimulus-driven saliency and orienting efficacy, as a function of the level of competition in the sensory input (Multi- vs. Lat-trials). For this, the two indexes were now included as video/trial-specific parametric modulators. This approach utilizes inter-stimulus variability to identify any region were the BOLD signal varied according to the lateralization of the stimulus-driven signals (co-variation with Sal_idx) or with the efficacy of the stimuli in attracting gaze/attention towards one hemifield (co-variation with Gaze_idx). For each run of each subject, the indexes were scaled to a 0–1 range before entering the fMRI analyses. The latter step was done so as to avoid comparing parameter estimates derived from predictors that included different amounts of variance (cf. gaze_idx in Lat-trials, with values always around +0.8 or −0.8; see Figure 1B, in green).

Separately for the two indexes, we re-estimated the first-level models now adding one regressor for each of the four conditions, cf. univariate model above. The additional regressors modeled the level of lateralized saliency/gaze-efficacy for each single trial (parametric modulators). For each condition, linear contrasts averaged the parameter estimates associated with the salience (or gaze) index across the two fMRI runs. At the group level, two separate analyses considered the Sal_idx and the Gaze_idx. Flexible factorial designs modeled four conditions of interest corresponding to the effect of Sal_idx (or Gaze_idx) separately in Lmulti, Rmulti, Llat, Rlat trials, plus the main effect of subjects. These models allowed us to test for regions were activity co-varied with salience (or gaze) differentially in videos with high vs. low competition (i.e., main effect “Multi > Lat”). The statistical thresholds were set to *p*-FWE-corr. = 0.05, cluster-level corrected for multiple comparison (cluster size estimated at *p*-unc. = 0.001).

## Results

### Overt Orienting Behavior

Spatial orienting behavioral data are shown in Figure 1B, where the ratio of the time spent in the two hemifields (Gaze_idx) is plotted against the corresponding saliency ratio (Sal_idx), separately for Lat- vs. Multi-trials and Left vs. Right-trials. Overall, the gaze data highlight that spatial orienting behavior in Lat- vs. Multi-trials was very different. The Lat-videos lead to a systematic shift of gaze towards the corresponding visual hemifield, with Gaze_idx values around ±0.8, irrespective of salience (see plots in green). By contrast, the Multi-videos lead to Gaze_idx values around zeros, indicating that the participants spent approximately the same amount of time fixating in the two hemifields (see plots in red). But notably, now saliency appeared to affect the spatial orienting behavior.

We formally assessed the relationship between gaze and saliency by correlating the two indexes, separately in the four experimental conditions. It should be noted that because these tests considered “left” and “right” trials separately, any “categorical” effect associated with the side of the main visual event on gaze orienting (cf. Lat-trials) did not contribute to the statistics reported here, which instead refer specifically to the effect of salience on orienting within-hemifield. The results showed that stimulus salience and the efficacy of the stimuli in orienting overt attention correlated significantly for the Rmulti-trials (*r* = 0.44, *p* = 0.014) and showed a statistical trend for the Lmulti-trials (*r* = 0.33, *p* = 0.074). For both trial-types, the largest the level of salience lateralization, the longer the time participants spent looking towards the most salient visual field (see Figure 1B, bottom panels in red). By contrast, when the videos included a single distinctive event, gaze was strongly lateralized on the side of the event and salience did not affect orienting (*r* = 0.10, *p* = 0.538 for Llat; *r* = 0.17, *p* = 0.302 for Rlat; see top panels in green).

Considering the presence of a rather wide age-range in our sample, we also tested for possible correlations between the saliency-gaze relationship and age, now using subject-by-subject variance. This did not reveal any significant correlation, but a statistical trend was found for the R-lat videos, where age correlated negatively with the relationship between saliency and gaze (*r* = −0.47; *p* = 0.069).

In addition to these analyses regarding the time spent within each hemifield, we sought to further characterize spatial orienting behavior by comparing the overall number of saccades in Multi- vs. Lat-trials and by assessing whether participants fixated salient locations/clusters with a different likelihood in Multi- vs. Lat-conditions. On average subjects made 1.9 saccades per second (±0.1, s.e.m.) when the stimulus contained a single lateralized event, while they made 2.4 saccades per second (±0.1, s.e.m.) during Multi-trials. A paired *t*-test formally confirmed the difference between the two stimulus conditions (*t*_(15)_ = 6.88, *p* < 0.001). The percentage of fixations falling inside the saliency clusters was larger in Lat- as compared to Multi-trials (37% vs. 32%, *T*_(138)_ = 2.0; *p* < 0.043), despite the number of these clusters was larger in Multi- as compared to Lat-videos (9.3 vs. 7.3, *T*_(138)_ = 4.0; *p* < 0.001). These additional results suggest that in Lat-trials the participants remained focused onto the most relevant event/object (fewer saccades, and more fixations falling within the saliency clusters), while in Multi-trials they shifted attention sequentially between the multiple distinctive events that characterized these high-competition videos (see also “Discussion” Section).

In sum, the gaze-data highlighted a qualitatively different orienting behavior in Lat- vs. Multi-trials, despite similar levels of saliency lateralization. This supports the notion that mechanisms other than pure bottom-up saliency contributed to spatial orienting during free viewing of these complex stimuli. Specifically, the participants tended to orient towards events displaying meaningful actions. In Lat-trials the presentation of a single, lateralized distinctive event governed orienting behavior (Figure 1B, in green). In Multi-trials there was high competition between multiple co-occurring events and now stimulus-driven salience was found to contribute to overt spatial orienting (see Figure 1B, in red).

### Overall Effect of Competition Between Visual Events

Before addressing the main issue concerning the competition between multiple events (i.e., “Multi vs. Lat”), for completeness we compared all four video-conditions vs. rest. This revealed the activation of a large portion of the cortex, including the entire occipital visual cortex, lateral and medial frontal regions, the insulae, medial temporal regions (comprising the hippocampus), the intraparietal sulcus (IPS) and the frontal eye fields (FEF), as well as the TPJ and the IFG. Because of the lack of specificity of this contrast we will not discuss these effects any further, aside briefly pointing out here that the activated areas included regions belonging to both the dorsal (IPS and FEF) and the ventral (TPJ and IFG) frontoparietal attention networks.

**Figure 2:**
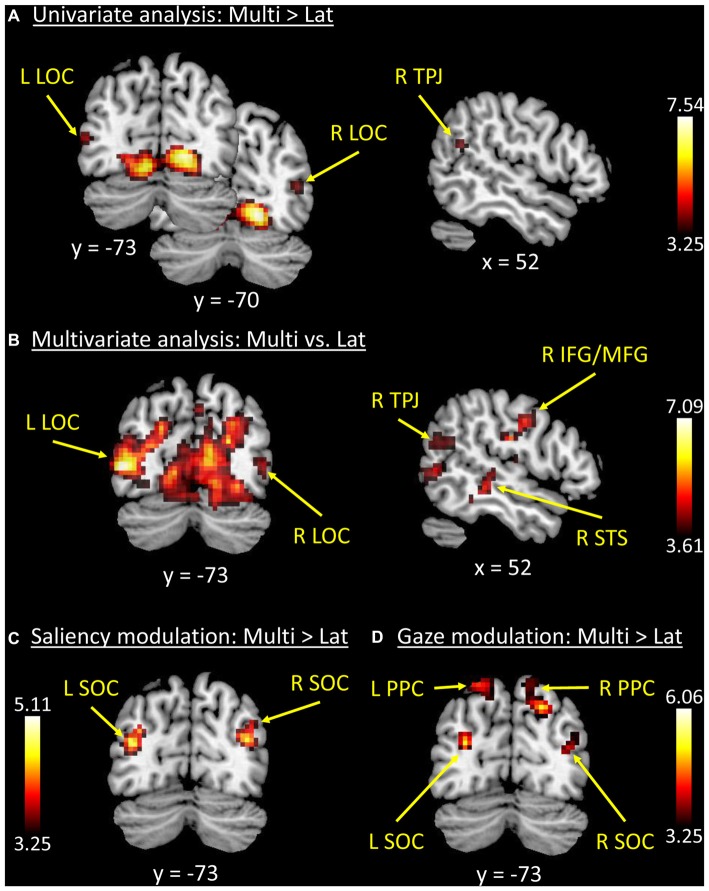
**Results of the whole-brain functional MR imaging (fMRI) analyses. (A)** Results of the univariate analysis comparing “Multi > Lat-trials”. These showed fully significant effects in occipital visual areas, plus a cluster in the right temporoparietal junction (R TPJ) that did not survive correction for multiple comparisons. **(B)** Results of the multivariate analysis (classification of “Multi vs. Lat” trials) that confirmed the effects in visual cortex, now also showing fully significant effects in the R TPJ and right inferior/middle frontal gyri (R IFG/MFG). **(C,D)** Trial-by-trial modulatory effects associated with the level of stimulus salience (Sal_idx, panel **C**) and orienting efficacy (Gaze_idx, panel **D**). Superior occipital regions plus the posterior parietal cortex (PPC) showed increasing BOLD responses with increasing levels of lateralization of salience and/or gaze, but more so on Multi-trials compared with Lat-trials (i.e., direct comparison between parametric modulators: “Multi > Lat”). See Tables 1, 2 for peak-coordinates and detailed statistics. Legend: L, left; R, right; Lat, lateralized trials; Multi, multiple trials; TPJ, temporoparietal junction; LOC, lateral occipital cortex; SOC, superior occipital cortex; IFG/MFG, inferior/middle frontal gyri; STS, superior temporal sulcus; PPC, posterior parietal cortex; x/y, coordinates in MNI space.

We tested for the influence of competition between distinctive events by directly comparing conditions with multiple vs. single-lateralized visual events. The univariate whole-brain analysis tested for “Multi > Lat”, averaging across left and right trials (see below for additional tests considering left and right trials separately). The results showed a significant cluster of activation comprising striate and extrastriate occipital visual cortex, extending into the ventral occipitotemporal cortex (see Figure 2A). A second significant cluster of activation was located in the precuneus (see Table 1).

The MVPA decoding revealed significant effects in the same regions, but with a larger cluster that now extended dorsally into the superior occipital cortex (SOC), and laterally including the lateral occipital cortex (LOC) and the R TPJ (see Figure 2B). Two additional significant clusters were found in the right superior temporal sulcus (R STS) and in the right inferior premotor cortex (R IFG/ middle frontal gyrus, RMFG; see Figure 2B, panel on the right). However, it should be noted that—albeit significant—the decoding accuracy for these two regions was just above chance level (see Table 1, rightmost column).

Accordingly, the MVPA results confirmed the results of the standard univariate analyses, but also highlighted several additional areas, in particular suggesting competition-related effects in regions belonging to the ventral attention system (i.e., R TPJ and R IFG/MFG). For completeness, we asked whether these differences between univariate and multivariate results merely reflected higher sensitivity of MVPA (Norman et al., 2006), or rather revealed some form of inter-digitated population coding within the ventral attention network (see also Silvetti et al., 2015). For this, we lowered the threshold of the whole-brain univariate analysis to *p*-unc. < 0.001, and looked for peaks around the regions observed in the MVPA. This revealed that R TPJ and bilateral LOC showed an effect for the contrast “Multi > Lat” (see Table 1, stats reported in italics; and Figure 2A). These additional tests indicate higher sensitivity of MVPA compared to standard univariate methods.

The lateralization of the TPJ findings in the right hemisphere prompted the question of whether this would hold for both left and right-trials, which were pooled in all the analyses above. Accordingly, we re-tested the effects of “Multi vs. Lat”, now considering left- and right-trials separately. For the univariate analysis, the two corresponding simple main effects showed analogous activations of the R TPJ (L-multi > L-lat: xyz = 63 −49 20, *T* = 3.28; R-multi > R-lat: xyz = 57 −40 14, *T* = 3.38; *p*-unc. < 0.001), without any effect in the L TPJ. The multivariate test that considered the left-trials only showed again an effect lateralized in the R TPJ (L-multi vs. L-lat: xyz = 48 −58 20, *T* = 3.72; *p*-unc. < 0.001), without any effect in the left hemisphere. The multivariate test with right-trials only revealed a robust effect in the R TPJ (xyz = 51 −55 14, *T* = 3.95, *p*-unc. < 0.001), but now also a weak effect in the left TPJ (xyz = −60 −58 17, *T* = 2.68, *p*-unc. < 0.01). Overall, these additional tests seem to suggest some dominance of the right TPJ for the processing of the videos including multiple events.

### Modulation According to Stimulus Salience and Orienting Efficacy

The analyses of the behavioral data highlighted a different contribution of stimulus-driven salience, depending on the level of competition present in the stimulus: salience was found to influence orienting behavior only when videos contained multiple competing events (see Figure 1B). We investigated the possible underlying neural basis of this condition-specific finding with two additional fMRI analyses. First, we tested for regions showing larger co-variation between the saliency lateralization index (Sal_idx) and the BOLD signal in Multi- compared with Lat-trials. This revealed a significant cluster of activation in the left SOC (see Figure 2C and Table 2). The corresponding region in the right hemisphere showed a statistical trend in the same direction (*p*-FWE-corr. = 0.079, corrected at whole-brain level). Thus, in these occipital regions the more salience was lateralized to one hemifield, the larger the BOLD response while viewing the video, but—critically—this effect was significantly larger in videos that included multiple competing events compared with videos with a single lateralized event.

**Table 2:** **Results of the analyses testing for activation associated with salience (Sal_idx) and gaze (Gaze_idx) indexes**.

	Region	Cluster		Peak	
		*p*	*k*	*t*	*x, y, z*
Sal_idx	L SOC	0.001	177	5.11	−39 −76 20
	*R SOC*	*0.079*	*66*	*4.47*	*39 −73 20*
Gaze_idx	L SOC	0.042	72	4.05	−24 −82 32
	*R SOC*	*0.132*	*50*	*4.74*	*45 −79 20*
	L PPC	<0.001	596	4.57	−24 −70 59
	R PPC			4.48	18 −67 59
	R PCN			4.37	9 −58 65
	L PCN			4.89	−15 −61 62

*Cluster: FWE-corrected p-value and cluster size (k = number of voxels). Peak: t-statistics and x, y, z-coordinates in MNI space. Regions: R/L, right/left hemisphere; SOC, superior occipital cortex; PPC, posterior parietal cortex; PCN, precuneus. In italics: the right LOC showed the relevant effects only at uncorrected p-values (p-unc < 0.001) and is reported because of the fully significant findings in the corresponding region of the left hemisphere*.

These results suggest that salience processing in these regions may occur only when stimulus-driven signals contribute to guide spatial orienting, that is, when videos include multiple events. We further explored this condition-specific link between brain activity and spatial orienting by considering the gaze lateralization index (Gaze_idx). It should be noted that in the relevant Multi-trials the Gaze_idx correlated with the Sal_idx (cf. behavioral results above, and Figure 1B, plots in red). Thus, testing for any BOLD co-variation with gaze may reveal similar results as using salience. Nonetheless, the gaze index should identify more specifically the Multi-trials where gaze/attention was *effectively* captured towards one side, a factor that we have previously shown to be critical for the activation of the dorsal attention system (Nardo et al., 2011). Indeed, the results of the co-variation analysis with Gaze_idx confirmed the modulation of activity in the SOC, but now also revealed a modulation of activity in the posterior parietal cortex (PPC, extending medially into the precuneus; see Figure 2D and Table 2).

## Discussion

The aim of this study was to investigate the role of stimulus-driven salience and competition between distinctive visual events during free-viewing of naturalistic stimuli, in the absence of any explicit goal-directed task. We expected that high-levels of competition would result in an increased demand of selective processing, which we hypothesized would increase the contribution of stimulus-driven salience to spatial orienting. Behaviorally, we found that indeed salience determined within-hemifield gaze orienting only in presence of multiple competing events. Functional imaging results showed that the processing of competing visual events engaged visual areas and the precuneus, but also the R STS and key nodes of the ventral attention network (R TPJ and R IFG/MFG, using multivariate analyses). Stimulus-driven salience was found to modulate activity in the SOC, selectively when the videos included multiple competing events. Moreover, the activation of the SOC as well as the PPC (in the dorsal attention network) co-varied with an index of spatial orienting efficacy. These results demonstrate that both dorsal and ventral attention systems contribute to the processing of competing events in naturalistic conditions, with a segregation between event detection and spatial selection in ventral and dorsal regions, respectively (cf. also Nardo et al., 2011).

The present study was designed to investigate spatial orienting as it takes place in life-like conditions similar to those we encounter in our everyday experience. Participants were asked to free-view short video-clips of dynamic real-world scenes, without any explicit task. These experimental settings are rather different from those used in most previous studies of visuospatial attention, where instead subjects are asked to actively engage in a specific task, such as searching for a target among many distractors. Thus, we make a distinction between “general attention control” and “goal-directed control”. The former would include all possible stimulus-related (external) influences, as well as many types of endogenous (internal) signals. Internal signals comprise both implicit information (such as that related to the recognition of semantically-relevant events in naturalistic conditions) and information that may be associated with an explicit task (e.g., task-relevant features defining a specific search template). A feature of the present study is that it did not involve any signal of the latter type: that is, there was no requirement of task-related, goal-directed control.

Our videos were characterized by the presence of either a single distinctive visual event lateralized in one hemifield or multiple events on both sides of space. The events included moving objects—in most cases people performing meaningful actions—that were semantically relevant, yet without being explicitly task/goal-related. This allowed us to ask the question of how the neural substrate traditionally involved in the selection of task-relevant stimuli (i.e., the frontoparietal attention networks, see Corbetta and Shulman, 2002) process such meaningful events, representative of real-world situations. Our approach partly relies on previous evidence that complex naturalistic stimuli can yield consistent patterns of brain activity, even in the absence of any explicit task (Hasson et al., 2004).

The standard univariate contrast that compared directly conditions with high vs. low competition (Multi- vs. Lat-trials) showed significant activation only within the occipital and occipitotemporal cortex, plus the precuneus. However, using a multivariate approach we showed that the level of competition was also represented in the R TPJ and the right inferior premotor cortex, that is, the two main nodes of the ventral attention network. Additional assessment of the imaging data revealed that the lack of significant effects in R TPJ and R IFG in the univariate analysis merely reflected the lower statistical power of the univariate compared to multivariate approach (see Table 1 showing that both regions activated at *p*-unc. < 0.001; cf. also Norman et al., 2006; Davis et al., 2014).

The R TPJ has been associated with stimulus-driven attentional reorienting, when a behaviorally relevant stimulus is presented outside the current focus of attention (Corbetta et al., 2008). Other studies have emphasized non-spatial aspects of attention control in R TPJ, including breaches/updating of expectations and responses to low frequency stimuli (Bledowski et al., 2004; Mulert et al., 2004; Geng and Vossel, 2013; for a proposal concerning R TPJ de-activation related to the filtering of spatial and non-spatial distractors, see also Shulman et al., 2009). Albeit enclosing substantial differences, these formulations emphasize the role of the ventral attention system in mediating the interaction between the current internal goals/expectations and the processing of the stimuli in the external environment (see Geng and Vossel, 2013; Macaluso and Doricchi, 2013; cf. also the notion of the R TPJ acting as a “circuit-breaker” in Corbetta and Shulman, 2002). By contrast, the current study did not involve any explicit task (see also above) and each trial included the presentation of a unique stimulus/video, which should minimize any task-based expectation and any need of updating thereof.

In a previous fMRI study that also involved passive viewing of complex and dynamic visual stimuli (i.e., a virtual visual environment), we found that the R TPJ activated on the presentation of unique, task-irrelevant events (moving avatars in that study; Nardo et al., 2011). The meaningful events engaged the R TPJ particularly when participants oriented gaze/attention towards these stimuli. This prompted us to suggest that the R TPJ activation reflected the transient engagement of a detection system, independent of task-relevance. Here, the high competition videos included multiple distinctive events, thus an explanation for the current findings would be that the Multi-trials lead to multiple, sequential detections with greater activation of the R TPJ as compared to the Lat-trials (single detection of just one distinctive event). In line with this, the eye-tracking data showed that participants made more saccades, and corresponding detections and shifts of spatial attention, in Multi- compared with Lat-trials (see also below, for a discussion of the possible confounding effect due to different number of saccades between conditions).

The current results are consistent with a previous fMRI study comparing bilateral vs. unilateral target-conditions using very simple visual stimuli (i.e., dots; see Beume et al., 2015). This study showed that, as compared to unilaterally-presented dots, bilaterally-presented dots were associated with increased activity in the right ventral attention network, including the R TPJ, the R IFG and perisylvian regions located in the temporal lobe (superior/middle temporal gyri). The authors suggested that the processing of bilateral events requires more attentional resources compared with unilateral processing, and that attentional regions were recruited to convey input from visual association areas to higher-order spatial representations that would integrate spatial signals arising in the two hemifields. Related to our proposal above, it should be noted that in this previous study the stimuli were presented for 400 ms and therefore, in bilateral trials, participants may have shifted attention between the two hemifields. However, the participants were asked to maintain central fixation during the detection task (covert orienting of attention) and no evidence with regard to this possibility was available.

Comparing conditions including bilateral vs. unilateral events is highly relevant for the understanding of spatial orienting deficits typically associated with right-hemisphere damage (unilateral spatial Neglect). Spatial Neglect is a complex neurological syndrome that include an orienting bias towards the ipsilesional (right) side of space, consistently reported in patients with lesions including the R TPJ (Corbetta and Shulman, 2002; Chechlacz et al., 2010; Ptak and Schnider, 2011). The deficit is exacerbated when patients are confronted with multiple stimuli presented across the two hemifields, that is, in conditions entailing high levels of competition (Riddoch and Humphreys, 1983; Bartolomeo and Chokron, 2001; Corben et al., 2001; Geng and Behrmann, 2006; Coulthard et al., 2008). Concurrent presentation of two stimuli in the two hemifields can lead to visual extinction of the stimulus presented on the contralesional side, again a deficit that has been associated with lesions of the R TPJ and neighboring regions in the posterior/superior temporal cortex (Friedrich et al., 1998; Karnath et al., 2003; Ticini et al., 2010; de Haan et al., 2012; Chechlacz et al., 2013; for related work using TMS in healthy subjects, see also Meister et al., 2006).

Our current results confirm the involvement of the right ventral attention network in the processing of competing events, with a lateralization to the right hemisphere that appeared to be largely independent of the stimulus-lateralization (cf. additional analyses that considered separately left and right trials). The eye-tracking data suggested that with the current stimulus material, which involve the presentation of meaningful events for a relatively long duration—as would happen in any everyday life situation—the high levels of competition in Multi-trials lead to the sequential detection of the distinctive events and shifting of spatial attention. Adding to previous findings using simple stimuli and explicit goal-directed tasks, here we demonstrate that the ventral attention system detects distinctive visual events within complex and dynamic visual scenes, even in the absence of any explicit goal-directed task. Moreover, unlike the few previous studies that showed activation of the R TPJ/R IFG for fully irrelevant stimuli (e.g., Asplund et al., 2010; Nardo et al., 2011), in the current study these effects can hardly be explained by low stimulus probability or some general effect of surprise. The finding that the R TPJ and R IFG engaged in the absence of any explicit goal-directed task poses a challenge for models of attention control that emphasize the role of task-set and/or expectations in the ventral attention system (Downar et al., 2001; Kincade et al., 2005; Geng and Mangun, 2011).

The main hypothesis of the current study was that stimulus-driven saliency would contribute to spatial orienting primarily in conditions entailing high levels of competition, when the presence of multiple visual events implies uncertain/conflicting information about the most relevant location in space. We predicted that in this situation the additional information provided by sensory salience would be most effective for guiding spatial orienting. Indeed, the behavioral results showed a correlation between saliency and gaze data only in Multi-trials (see Figure 1B, plots in red). By contrast, in the Lat-trials the subjects oriented their gaze systematically towards the distinctive events and there was no effect of salience (despite the good amount of Sal_idx variability also in these trials; cf. x-axis in the top-panels of Figure 1B). These behavioral data indicate that in Lat-trials the distinctive events governed spatial orienting over and above any bottom-up influence, whereas the latter did contribute to spatial orienting in presence of multiple, competing events.

The analyses of imaging data sought to identify where in the brain this condition-specific influence of saliency on spatial orienting was implemented. The results showed that both stimulus salience (Sal_idx) and stimulus efficacy (Gaze_idx) modulated activity in the SOC, selectively in high competition Multi-trials (see Figures 2C,D). In addition, the index related to the stimulus orienting efficacy was found to modulate responses of the PPC, in the dorsal attention network (Corbetta and Shulman, 2002).

These results are in agreement with previous findings that demonstrated coding of salience in PPC and—more specifically—that response of the dorsal attention network reflects the efficacy of these stimulus-driven signals for spatial orienting and selection (Bogler et al., 2011; Nardo et al., 2011, 2014; Santangelo and Macaluso, 2013). Here, the engagement of these dorsal regions provide us with a possible physiological substrate for the behavioral finding that salience affected orienting behavior selectively in high competition trials. Beyond the classical view that the dorsal attention system is associated with goal-directed attentional and oculomotor control (Corbetta and Shulman, 2002; Müri, 2006; Corbetta et al., 2008), recent evidence emphasizes the integration of multiple control signals for spatial selection in the PPC (see also Macaluso and Doricchi, 2013). The PPC is sensitive to bottom-up attentional influences associated with stimulus salience (Geng and Mangun, 2009), contains attention maps indexing space to support the selection of multiple objects at the same time (Somers and Sheremata, 2013), and is implicated in feature binding, but only when spatial information is available to resolve ambiguities about the relationships between object features (Shafritz et al., 2002). These findings are consistent with the notion that PPC contains topographical representations of the visual space that code for the relative relevance of different locations in the environment (“saliency maps”: Gottlieb et al., 1998; Gottlieb, 2007; Bogler et al., 2011; Nardo et al., 2011), and that integrate bottom-up and top-down signals for the control of spatial orienting (see also, “priority maps”: Bisley and Goldberg, 2010; Arcizet et al., 2011; Ptak, 2012).

Our results implicate both dorsal and ventral attention systems during spatial orienting in naturalistic viewing conditions that included multiple competing events. The notion that spatial orienting relies on the interplay between the two frontoparietal control systems is well acknowledged in the literature (e.g., Corbetta and Shulman, 2002; Macaluso, 2010; Vossel et al., 2014). This interplay has been studied primarily in tasks entailing explicit goal-directed attention (such as spatial cueing tasks), leading to the proposal that the ventral system acts as a “circuit breaker” that re-sets top-down control that the dorsal system exerts on visual areas (Corbetta and Shulman, 2002). Subsequent investigations further specified the constraints that can lead to the engagement of the ventral network, including stimulus saliency/novelty (Downar et al., 2000, 2001, 2002; Bledowski et al., 2004; Mavritsaki et al., 2010), task-relatedness (Kincade et al., 2005; Corbetta et al., 2008; Geng and Mangun, 2011) and contextual updating (Geng and Vossel, 2013). Our current data do not provide us with any information about the dynamics of the interaction between the two attention networks (Vossel et al., 2012), but indicate that both systems engage even in the absence of any explicit goal-directed task. We propose that here the ventral system detects the occurrence of multiple competing events, signaling to the dorsal (posterior parietal) system that salience needs to be taken into account in order to resolve the competition and select the spatial location with the highest priority (cf. McMains and Kastner, 2011; for related proposal concerning selective processing in the visual cortex). Future studies may use TMS and/or analyses of effective connectivity to shed light on the temporal sequence of the activation of ventral and dorsal frontoparietal regions during free-viewing of naturalistic stimuli.

While the use of complex stimulus material and free-viewing provide us with experimental settings that approximate attention control in the real world, it should be acknowledged that this approach entails also several limitations. First, here the definition of what constitutes a distinctive event, and thus whether a specific video was classified as a Multi- or Lat-trial, had to be relatively arbitrary. We sought to select a pool of videos where there was a clear difference between the distinctive foreground event/s and the scene background. On average, the eye-tracking data confirmed that there was indeed a measurable, objective difference between the videos assigned to the different conditions (see Figure 1B). This was further supported by the different number of saliency clusters in Multi- vs. Lat-videos, as well as by the different percentage of fixations falling inside the saliency clusters in the two conditions (see “Results” Section). Taken together, these data indicate that indeed there was a systematic difference between the videos selected for the two conditions. Nonetheless, it is possible that for some specific video the condition-assignment (or inclusion in the final pool of videos) could have been done in a different way.

Second, the different conditions lead to different overt motor behavior (e.g., number of saccades), which is a limitation for the interpretation of the imaging results. Indeed, the large activation of visual areas in both univariate and multivariate analyses (including the striate cortex) is most likely due to differences in the visual input arriving to the occipital visual cortex, as a consequence of the different oculomotor behavior. Nonetheless, in previous studies we have explicitly addressed the impact of overt eye-movements on the pattern of activity associated with event-detection and salience-processing by scanning participants also during covert viewing of the stimuli (Nardo et al., 2011, 2014). By directly comparing overt vs. covert viewing conditions we found that while activity in early visual areas was indeed strongly dependent on eye-movements, the pattern of activation in R TPJ, R IFG and PPC, as well as extra-striate visual cortex, were largely unaffected by overt/covert viewing condition. Thus, we expect that also the current findings about the effect of competition between events and the interaction between this and salience processing does not merely reflect a motor-related confound. This view is also supported by the current finding that in PPC there was a positive co-variation between BOLD response and Gaze_idx: that is, activity in PPC increased for those Multi-videos that led to some gaze-lateralization. High gaze-lateralization means that the participants spent longer time looking towards a specific hemifield. In general, this is associated with a decrease (rather than an increase) of the number of saccades. For instance, the comparison between Lat- vs. Multi-trials, which are associated with high vs. low Gaze_idx respectively (cf. Figure 1B), showed that the number of saccades was indeed smaller in the former (1.9 vs. 2.4 saccades/s, see “Results” Section). Accordingly, it seems unlikely that the positive co-variation between the BOLD signal in PPC and Gaze_idx can be attributed to some increase in eye movements.

Third, besides the differences in visual complexity and number of saccades, Lat- and Multi-trials may differ in the number of “interesting” or “relevant” objects. While this issue could in principle be addressed using manual object-labeling (e.g., see “labelme” project; Russell et al., 2008), it should be pointed out that the stimuli presented in the current study included a total of 5250 frames, which makes any such manual labeling procedure unfeasible. Instead, here we opted for a careful selection of the videos and the computational analysis of the stimuli (saliency maps), which appeared suitable to achieve the current aim of studying the interplay between event-competition and bottom-up saliency. Moreover, it should be noted that the computational approach has the advantage that it can be easily applied to any new set of stimuli, thus facilitating future research based on the current results.

In conclusion, this study was aimed at investigating how competition between distinctive visual events and stimulus-driven salience affect spatial orienting, when viewing naturalistic stimuli without any goal-directed task. We found that salience contributed to spatial orienting only when the level of competition was high (Multi-trials). While in situations of low competition (Lat-trials) spatial orienting was driven by the main visual event, the presence of multiple competing events implicated an additional role of stimulus-driven signals for the selection of the most relevant spatial locations. The imaging analyses highlighted the engagement of the right ventral attention system (R TPJ and R IFG) for the processing of highly competing stimuli. We relate the increased activation of the ventral system with the detection of multiple events in the high competition trials and sequential shifting of spatial attention between these events. Selectively for the high competition trials, we found that salience modulated activity in the SOC and that the efficacy of the stimuli for spatial orienting modulated the same occipital areas, as well as the PPC. We link these effects in dorsal regions with the representation of bottom-up signals that effectively contribute to spatial orienting (Gottlieb, 2007; Bogler et al., 2011; Nardo et al., 2011). We conclude that dorsal and ventral frontoparietal attention networks play specific roles during the processing of competing events and spatial orienting in life-like conditions. Our results contribute to the emerging field of neuroimaging studies that try to characterize mechanisms of attention control relevant for brain functioning in the real world (Peelen and Kastner, 2014; see also Hasson et al., 2004).

## Author Contributions

DN, CR and EM designed the study. DN created the stimuli, ran the experiments and analyzed behavioral data. DN, CR and PC analyzed fMRI data. DN and EM wrote the manuscript. DN, PC, CR and EM edited the manuscript.

## Conflict of Interest Statement

The authors declare that the research was conducted in the absence of any commercial or financial relationships that could be construed as a potential conflict of interest.

## Footnotes

^1^http://www.coloribus.com

^2^http://www.slneuroimaginglab.com/mt-tools

^3^http://www.smivision.com/en/gaze-and-eye-tracking-systems/products/redm.html

^4^http://www.slneuroimaginglab.com/mt-tools

^5^https://sites.google.com/site/tdtdecodingtoolbox
